# Changes in Intrapersonal Factors of Participants in the Pregnancy Remote Monitoring Study Who Are at Risk for Pregnancy-Induced Hypertension: Descriptive Quantitative Study

**DOI:** 10.2196/42686

**Published:** 2023-09-06

**Authors:** Dorien Lanssens, Thijs Vandenberk, Valerie Storms, Inge Thijs, Lars Grieten, Lotte Bamelis, Wilfried Gyselaers, Eileen Tang, Patrick Luyten

**Affiliations:** 1 Limburg Clinical Research Center/Mobile Health Unit Faculty of Medicine and Life Sciences Hasselt University Hasselt Belgium; 2 Department of Obstetrics and Gynaecology Ziekenhuis Oost-Limburg Genk Belgium; 3 Faculty Medicine and Life Sciences Department of Physiology Hasselt University Diepenbeek Belgium; 4 Centre for Translational Psychological Research TRACE Ziekenhuis Oost Liburg Genk Belgium; 5 Faculty of Psychology and Educational Sciences KULeuven Leuven Belgium; 6 Faculty of Psychology and Educational Sciences Vrije Universiteit Brussel Brussel Belgium; 7 Educational and Health Psychology Research Department of Clinical University College London London United Kingdom

**Keywords:** intrapersonal factors, peripartum period, pregnancy, pregnancy-induced hypertension, remote monitoring

## Abstract

**Background:**

The peripartum period, defined as the period from the beginning of the gestation until 1 year after the delivery, has long been shown to be potentially associated with increased levels of stress and anxiety with regard to one’s transition to the status of parent and the accompanying parental tasks. Yet, no research to date has investigated changes in intrapersonal factors during the peripartum period in women at risk for pregnancy-induced hypertension (PIH).

**Objective:**

The aim of this study is to explore and describe changes in intrapersonal factors in participants at risk for PIH.

**Methods:**

We used an explorative design in which 3 questionnaires were sent by email to 110 participants the day following enrollment in the Pregnancy Remote Monitoring program for pregnant women at risk for PIH. Women were invited to complete the questionnaires at the beginning of their participation in the Pregnancy Remote Monitoring project (mostly at 14 weeks of gestation) and after approaching 32 weeks of gestational age (GA). The Generalized Anxiety Disorder-7 Scale (GAD-7) and the Patient Health Questionnaire-9 were used to assess anxiety and depression, and adaptation of the Pain Catastrophizing Scale was used to measure trait pain catastrophizing.

**Results:**

Scores were significantly higher at 32 weeks of GA than at the moment of enrollment (GAD-7 score=7, range 4-11 vs 5, range 3-8; *P*=.01; and Patient Health Questionnaire-9 score=6, range 4-10 vs 4, range 2-7; *P*<.001). The subscale scores of the Pain Catastrophizing Scale were all lower at 32 weeks of GA compared with 14 weeks of GA (rumination: 4, range 1-6 vs 5, range 2-9.5; *P*=.11; magnification: 3, range 1-5.5 vs 4, range 3-7; *P*=.04; and helplessness: 5, range 2-9 vs 6, range 3.5-12; *P*=.06). The proportion of women with a risk for depression (GAD-7 score >10) was 13.3% (10/75) at enrollment and had increased to 35.6% (26/75) at 32 weeks of GA.

**Conclusions:**

This study shows that pregnant women at risk for PIH have higher levels of stress and anxiety at 32 weeks of GA than at the moment of enrollment. Further research is recommended to investigate potential strategies to help pregnant women at risk for PIH manage feelings of stress and anxiety.

**Trial Registration:**

ClinicalTrials.gov NCT03246737; https://clinicaltrials.gov/study/NCT03246737

## Introduction

The peripartum period, defined as the period from the beginning of gestation until 1 year after delivery, has long been shown to be potentially associated with increased levels of stress and anxiety with regard to one’s transition to the status of parent and the accompanying parental tasks [1]. It is normal for expectant or new mothers to report anxiety and stress symptoms. Up to two-thirds of women experience worries, most commonly consisting of fear about having an abnormal baby; complications during pregnancy or birth; their ability to care for the baby, including breastfeeding and soothing the infant when crying; as well as concerns about body changes, partner relationships, job performance, finances, cleanliness, etc [2]. Elevated untreated perinatal anxiety may negatively impact maternal health [3-5]; the child’s cognitive [6], emotional, and behavioral development [7]; and the mother-infant relationship [8]. Given this, the detection of problematic anxiety through the use of an effective screening tool may be important in screening for risk of problematic anxiety, prevention, early intervention, and treatment in the field of perinatal mental health [9]. Despite the fact that heightened maternal preoccupation in the perinatal period proved to have an adaptive function, particular physical and somatic at-risk circumstances of pregnancy may potentially fuel already increased levels of stress and anxiety to the extent of becoming maladaptive or psychopathological [10,11].

A medically high-risk pregnancy is defined as “any pregnancy in which there is evidence of an actual or potential threat of harm to the life or health of the mother or the baby because of a disorder or situation coincidental with or unique to pregnancy” [12]. Health conditions that lead to increased risk can be obstetrical (eg, placenta previa), maternal (eg, preeclampsia), or fetal (eg, prematurity) [13]. These complications affect approximately 15%-20% of pregnancies annually [14,15]. Medical complications impact not only the health and well-being of the mother but also her developing infant (and the entire family) [16]. Aside from the physical implications, a medically complicated pregnancy can also be a source of significant stress and anxiety for mothers [3,17,18]. Medical complications of pregnancy can invoke stressful emotional pre- and postpartum responses in parents which differ from the usual stress associated with parenthood [19]. In particular, medical complications give rise to feelings of lack of control, worry about the fetus, and uncertainty regarding pregnancy outcomes, which add to women’s distress and anxiety levels [20]. A recent study has shown that women experiencing a medically high-risk pregnancy have a 5.17-times higher incidence of anxiety disorders compared to women experiencing a medically low-risk pregnancy [21,22]. The extant literature indicates that anxiety among women experiencing a medically complicated pregnancy is an important area of investigation [16].

Pregnancy-induced hypertension (PIH) complicates between 6% and 10% of all pregnancies and can turn a low-risk pregnancy into a high-risk pregnancy. It is defined as an in-office measurement with a systolic blood pressure (BP) above 140 mm Hg and a diastolic BP above 90 mm Hg. Severe range BP is above 160 mm Hg systolic and 110 mm Hg diastolic. PIH refers to one of four conditions: (1) preexisting hypertension, (2) gestational hypertension, (3) preeclampsia, and (4) unclassified hypertension. PIH is one of the leading causes of maternal morbidity and mortality. Hypertensive disorders may result in fetal complications such as growth restriction, oligohydramnios, placental abruption, preterm birth, and perinatal death [23,24]. The usual care for women with pregnancies complicated by PIH comprises clinical follow-up, serological investigation, and fetal ultrasound evaluation. The type and frequency of follow-up depends on the nature and severity of the hypertensive disorder [23]. The goal of treatment is to prevent significant cerebrovascular and cardiovascular events in the mother without compromising fetal well-being [25]. As explained before, the peripartum period has long been known to be associated with increased levels of stress and anxiety related to the transition to parenthood and parental tasks and concerns associated with this transition [1]. However, when PIH is present, it potentially increases the already elevated levels of stress and anxiety associated with this normative and normally adaptive heightened “maternal preoccupation” in the perinatal period [10,11]. In this context, cognitive factors, such as catastrophizing or rumination, insecure attachment styles, and personality factors, such as perfectionism and dependency, have been associated with problems in negotiating the challenges of parenthood, which are expressed as increased levels of anxiety and depression [26-28].

New techniques to support these strategies have recently been developed, including remote monitoring (RM), which can broadly be defined as the use of telecommunication technologies to facilitate the transmission of medical information and services between health care providers and participants [29]. RM is a relatively new approach that facilitates patient management at home [30]. As part of the Limburg Clinical Research Center in collaboration with Hasselt University, Ziekenhuis Oost-Limburg (Genk), a large hospital in the east of Belgium, added RM to the usual prenatal care of women with PIH. This project is called the Pregnancy Remote Monitoring (PREMOM) project. A previous publication explored the role of participants’ psychosocial characteristics (severity of depression or anxiety, cognitive factors, attachment styles, and personality traits) in their adherence to RM. The study demonstrated the relationships between adherence to RM and patient characteristics in women at risk of PIH. Alertness toward the group of women who show less than optimal adherence is essential. These findings call for further research on the management of PIH and the importance of individual tailoring of RM in this patient group [31]. In the ongoing PREMOM II study (a multicentric randomized controlled trial [32]) is this rationale included for further investigation on a larger sample size. The first results are expected in 2024.

In developing those new strategies (including RM) to optimize the obstetric care of pregnant women at risk for PIH, it is important to investigate patient characteristics and intrapersonal factors. To the best of our knowledge, no research to date has been conducted about changes in the intrapersonal factors during the peripartum period in women at risk for PIH. Therefore, the primary aim of this study was to explore potential changes in anxiety and depression during pregnancy, from the moment of enrollment in the PREMOM I study (at, on average, 14 weeks of gestational age [GA]) to 32 weeks of GA. Our hypothesis was that anxiety and depression would have increased by 32 weeks of GA compared to the moment of enrollment due to the fact that delivery and associated complications are imminent. The secondary aim of this study was to study the level of catastrophizing at both measurement moments. We hypothesized that the levels of catastrophizing would increase between the inclusion enrollment and 32 weeks of GA due to pregnancy-related symptoms.

## Methods

### The PREMOM I study

**Figure 1:**
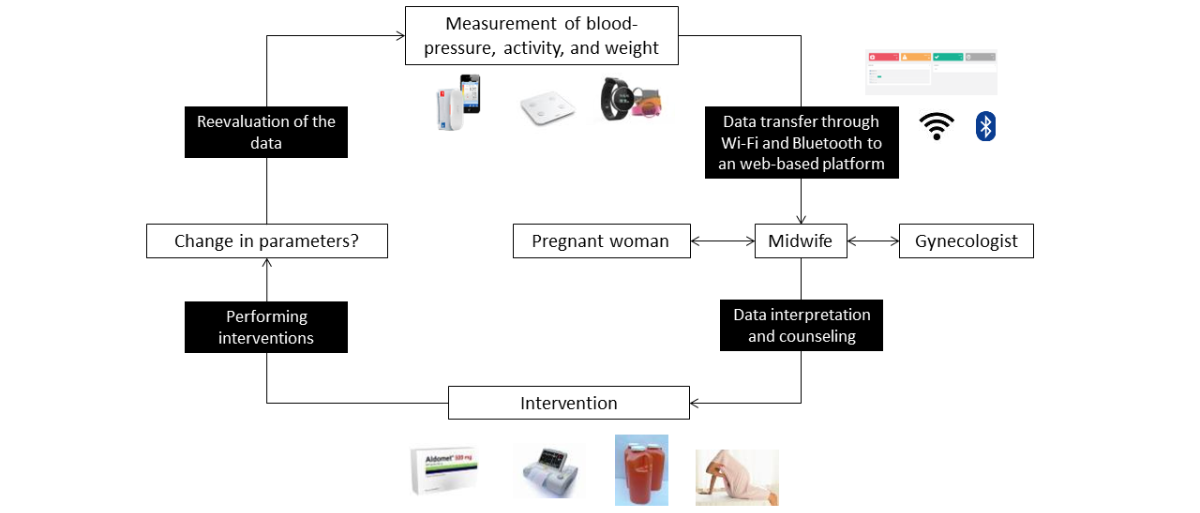
Workflow of the PREMOM I study. PREMOM: Pregnancy Remote Monitoring.

The PREMOM I study was set up in January 2015 as a collaboration in Belgium between Hasselt University, Ziekenhuis Oost-Limburg (Genk), and 7 other hospitals (AZ Vesalius, Tongeren; Heilig Hart Ziekenhuis, Mol; JESSA, Hasselt; Maria Ziekenhuis Noord Limburg, Overpelt; Sint Franciskusziekenhuis, Heusden; S. Trudo, Sint Truiden; and Ziekenhuis Maas & Kempen, Maasmechelen). Women who participated in the PREMOM I study received obstetric surveillance through a BP monitor, an activity tracker, and a weight scale. They were asked to perform 2 BP measurements each day (morning and evening), to wear the activity tracker continuously, and to register their weight once a week in the app. This information was to be recorded until the moment of their delivery or until they were admitted to the hospital. The data collected using these devices were transferred to a web-based dashboard developed by the Mobile Health Unit (Limburg Clinical Research Center, Hasselt University; Ziekenhuis Oost-Limburg; Jessa). Predefined alarm signals were developed. Alarm signals were communicated to the responsible obstetrician so that treatment options could be agreed upon with the midwife before the pregnant woman was contacted. The therapeutic interventions were in line with local treatment procedures. The workflow is summarized in Figure 1.

In the past few years, our research team has published several articles related to the PREMOM I study that demonstrated the benefit of RM for pregnant women at elevated risk of developing PIH. Two articles made comparisons between women who received RM and women who had an increased risk of developing PIH but did not participate in the PREMOM I study (conventional care group and control group [CG]) based on a retrospective and observational design. In both studies, prenatal hospitalization to a prenatal ward (until the moment of delivery), diagnosis of preeclampsia, and number of inductions were reduced in the RM group compared with the CG. However, women in the RM group had significantly higher risks of developing gestational hypertension and a spontaneous start of the birth process compared with the CG. In the study conducted in 2015, the total number of neonatal hospitalizations in the neonatal intensive care unit was lower in the RM group than in the CG group; these findings were not confirmed in the study conducted in 2015-2016. In the later study, the total number of prenatal visits was lower in the RM group than in the CG group; this difference was not apparent in the earlier study [33-35].

### Participants

**Figure 2:**
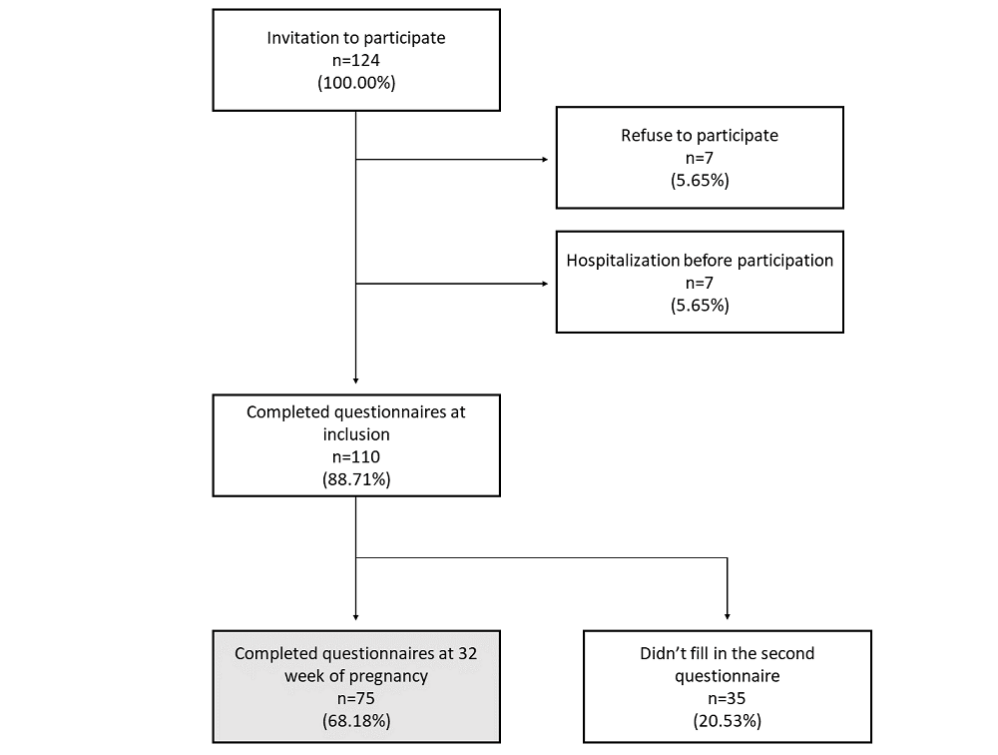
Patient enrollment.

The process of patient enrollment is shown in Figure 2. A total of 124 pregnant women from the PREMOM study were invited to participate in this study. Seven (5.65%) of them refused to participate because of a lack of interest. Of the remaining 117 pregnant women, 7 (5.65%) were hospitalized at the prenatal ward due to complications before they could fill out the questionnaires. In total, 110 pregnant women (response rate: 88.71%) completed the questionnaires at the start of their participation in the PREMOM project. At 32 weeks of GA, 75 of them (retention rate: 68.18%) also filled out the questionnaires.

### Data Collection

For this study, an explorative and descriptive quantitative design was used. Pregnant women were given written information about the study when they received their BP monitor and activity tracker at the start of their RM program. The following day, all participants received an email containing a SurveyMonkey link. After logging in, the participants were asked to complete 3 questionnaires (see subsection Questionnaires) at 2 moments in time: the day following their enrollment in the RM program and at 32 weeks of GA. Demographic and obstetric characteristics of the participants were collected at the moment of enrollment in the PREMOM program and after delivery, through the hospital administration or billing records.

### Questionnaires

The Generalized Anxiety Disorder-7 scale (GAD-7) was used to detect anxiety disorders. The GAD-7 was developed within the classical theory as a brief, dimensional screening instrument that aims at identifying probable cases of anxiety disorder and also assessing symptom severity [36]. Simpson et al [37] stated that the GAD-7 represents a clinically meaningful instrument for screening in a perinatal population. The GAD-7 consists of 7 items to be rated on a balanced 4-point Likert scale ranging from 0 (“not at all”) to 3 (“almost every day”). In addition, the Patient Health Questionnaire-9 (PHQ-9) [38] was used to measure the severity of depression. The PHQ-9 was developed for use in primary care settings and has been extensively tested for validity among diverse populations [39]. Nine questions were asked to be scored on a 4-point Likert scale ranging from 0 (not at all) to 3 (almost every day). Finally, the Pain Catastrophizing Scale (PCS) is used to assess the severity of worry in the context of actual or anticipated pain. It conceptualizes catastrophizing as a multifaceted construct with 3 dimensions, each with a separately summed score: rumination (“I can’t stop thinking about how much it hurts”), magnification (“I worry that something serious may happen”), and helplessness (“There is nothing I can do to reduce the intensity of the pain”). The PCS consists of 13 items, which query the 3 subscales to be rated on a balanced 5-point Likert scale ranging from 1 (“not applicable at all”) to 5 (“fully applicable”) [40]. The scores are calculated for each subscale, and the total scores are used. This questionnaire has been adapted by the research team to refer to pregnancy-related questions. For all 3 questionnaires, higher scores indicate higher levels of feelings of depression, anxiety, and catastrophizing, respectively.

### Statistical Analysis

Data analysis was performed using R statistical software (version 3.2.2; R Foundation). No data imputation was performed for missing data. The study sample size was based on an a priori sample size calculation with a power of minimum 70 participants. The Shapiro-Wilk test was used to assess whether or not the data were normally distributed. Nonparametric tests were used in case where the normality assumption was violated. Skewed data are expressed as a median and IQR. Independent *t* tests (parametric) or Mann-Whitney *U* tests (nonparametric) were used for between-group comparisons. Both dimensional and categorical analyses were performed. For the PCS, catastrophizing was dichotomized as described by Olsson et al [41]. The 2 categories are labeled as “noncatastrophizing” (≤17 for the total sum) and “catastrophizing” (>17 for the total sum). A score of more than 38 is classified as “clinically relevant.” *P* values <.05 were considered statistically significant.

### Ethical Considerations

The study was approved by the Ethical Committee of the Ziekenhuis Oost-Limburg (15/093U - B371201526777) and all participants provided written informed consent. The study was also registered at ClinicalTrails.gov (NCT03246737).

## Results

### Participants Demographics

In total, 75 participants completed all questionnaires at both time points. Patient mean age was 30 (28.0-32.5) years, and 28 (37.3%) of the women were pregnant for the first time. Table 1 provides detailed information about the demographics of the study participants.

**Table 1:** Demographics of the study participants (N=75).

	Participants
Age (years), mean (SD)	30 (28-32.50)
Prepregnancy weight (kg), mean (SD)	79.97 (18.78)
Height (cm), mean (SD)	166.9 (7.1)
BMI (kg/m^2^), mean (SD)	27.68 (23.84-31.87)
Primigravida, n (%)	28 (37.33)

### The Evolution of Anxiety, Depression, and Level of Catastrophizing During Pregnancy

The results (Table 2) showed that the levels of anxiety, depression, and several aspects of catastrophizing changed statistically over time. Pregnant women at 32 weeks of GA feel more anxiety and depression than women at the moment of enrollment (GAD-7 score=7, range 4-11 at 32 weeks of GA vs 5, range 3-8; *P*=.01 at the moment of enrollment; and PHQ-9 score=6, range 4-11 at 32 weeks of GA; and 4, range 2-7; *P*<.001 at the moment of enrollment). Only magnification was significantly lower at 32 weeks of GA compared to the moment of enrollment (3, range 1-5.5 at 32 weeks vs 4, range 3-7; *P*=.04 at the moment of enrollment).

**Table 2:** Overview of study results.

			Enrollment (N=75), median (IQR)		32 weeks (N=75), median (IQR)		*P* value
**Anxiety and depression**							
	GAD-7^a^	5 (3-8)		7 (4-11)		.01^b^	
	PHQ-9^c^	4 (2-7)		6 (4-10)		<.001^d^	
**Catastrophizing–PCS^e^ scale**							
	Rumination	5 (2-9.5)		4 (1-6)		.11	
	Magnification	4 (3-7)		3 (1-5.5)		.04^b^	
	Helplessness	6 (3.5-12)		5 (2-9)		.06	

^a^GAD-7: Generalized Anxiety Disorder scale.

^b^*P*<.05.

^c^PHQ-9: Patient Health Questionnaire.

^d^*P*<.001.

^e^PCS: Pain Catastrophizing Scale.

In Table 3 the number of participants in each anxiety and depression category is shown. Based on the GAD-7, 10 (13.33%) pregnant women of the 75 were clinically anxious (score≥10) at the time of enrollment and 26 (34.67%) of the 75 at 32 weeks of GA. On the PHQ-9 questionnaire, 7 (9.33%) of the 75 pregnant women were clinically depressed (score≥10) at the time of enrollment and 22 (29.33%) of the 75 at 32 weeks of GA.

**Table 3:** Overview of participants in questionnaire category.

		Enrollment (N=75), n (%)		32 weeks GA^a^ (N=75), n (%)
**GAD-7^b^**				
	0-4	37 (49.33)	22 (29.33)	
	5-9	28 (37.33)	27 (36)	
	10-14	5 (6.67)	20 (26.67)	
	15-21	5 (6.67)	6 (8)	
	Clinically not anxious (score <10)	65 (86.67)	49 (65.33)	
	Clinically anxious (score ≥10)	10 (13.33)	26 (34.67)	
**PHQ-9^c^**				
	0-4	44 (58.67)	27 (36)	
	5-9	24 (32)	26 (34.67)	
	10-14	4 (5.33)	18 (24)	
	15-21	3 (4)	45 (33)	
	Clinically not anxious (score <10)	68 (90.67)	53 (70.67)	
	Clinically anxious (score ≥10)	7 (9.33)	22 (29.33)	

^a^GA: gestational age.

^b^GAD-7: Generalized Anxiety Disorder-7 scale.

^c^PHQ-9: Patient Health Questionnaire-9.

Based on the PCS, 25 (33.33%) pregnant women of the 75 crossed the cutoff value of more than 17 for the total sum described by Olsson et al [41] at the time of enrollment. Three (4%) of the 75 pregnant women scored more than 38 and are classified as clinically relevant. At 32 weeks of GA, 25 (33.33%) of the 75 pregnant women scored more than 17, while 4 (5.33%) of the 75 pregnant women had a higher score than 38 (Table 4).

**Table 4:** Overview of catastrophizing on the Pain Catastrophizing Scale (PCS; N=75).

	Noncatastrophizing (≤17), n (%)	Catastrophizing (>17), n (%)	Clinically relevant (>38), n (%)
Enrollment	50 (66.67)	25 (33.33)	3 (4)
32 weeks GA^a^	50 (66.67)	24 (32)	4 (5.33)

^a^GA: gestational age.

## Discussion

We sought to explore and describe potential changes in some intrapersonal factors of participants at risk for PIH. To the best of our knowledge, this is the first publication targeting this change in psychological factors during pregnancies at risk of this complication. Two interesting sets of findings emerged.

### Principal Results

First, in line with our expectations, scores on the GAD-7 and PHQ-9 were higher at 32 weeks of GA compared to the moment of enrollment. The scores on the subscales of the PCS were all lower at 32 weeks of GA compared with 14 weeks of GA. Regarding the primary end point (higher score for anxiety and depression at 32 weeks compared to 14 weeks), this hypothesis can be adapted. This study showed that pregnant women with PIH followed a normal pattern of increased pregnancy scores, as described by Biaggi et al [42]. The proportion of pregnant women identified by the GAD-7 with a risk for anxiety (GAD-7 score >10) was 13.3% (10/75) at enrollment and had risen to 35.6% (26/75) during 32 weeks of GA. This is higher than the reported 8.5% of pregnant women surveyed in the GAD-7 by Sutter-Dalay et al [28] and the 9.9% prevalence described by Melville et al [43]. Additionally, the scores on the PCS for pregnant women at risk of PIH are in line with those of women experiencing a normal pregnancy; for both women with and without a risk for the development of PIH, the risk for anxiety and depression is low at the onset of the pregnancy compared to the end of the pregnancy [44].

Regarding the second research aim of this study, catastrophizing in women included in this study decreased significantly for the subscale “magnification,” but was relatively stable for the subscales “rumination” and “helplessness,” and for the categorical approach. This is in line with the reported stability of catastrophizing over time during a normative pregnancy [40]. However, the study of Ollson et al [41] reported that levels of catastrophizing changed (both increased and decreased) over time in a minority of participants. So, instead of the stable PCS score for the subscale “magnification” for normal pregnancy, pregnant women with a PIH have a significantly reduced score on this subscale.

### Pregnancies Complicated With PIH Compared to Low-Risk Pregnancies

#### GAD-7

For the GAD-7, Zhong et al [45] reported that 14 of 946 (1.48%) of the pregnant women in their study were classified as having anxiety at the moment of enrollment (GA<16 weeks). In late pregnancy (GA≥28 weeks), Yu et al [46] stated that the prevalence of the classification of anxiety was 7.9%. Both those studies showed a lower prevalence of the classification of anxiety than the prevalence of the classification of anxiety in our study (10/75, 13.33%) at the moment of enrollment and 34.67% (26/75) at 32 weeks of GA.

#### PHQ-9

At 16 weeks of GA, 13.71% (159/1160) of the study population in the study of Avalos et al [47] were diagnosed as clinically depressed, which is almost 5% more than observed in our study at the moment of enrollment (7/75, 9.33%). In later pregnancy, the study of Gallis et al [48] reported a higher prevalence of women diagnosed as clinically depressed in their third trimester compared to our study population (570/1154, 49.39% vs 22/75, 29.33%, respectively). This is a comparison to the study of Yu et al [46], where the prevalence of depressive symptoms in late pregnancy (GA≥28 weeks) was 20% lower compared to the pregnant women in our study at 32 weeks of GA (75/813, 9.2%; 95% CI 7.2%-77.2% vs 22/75, 29.33%). Finally, participants who were in their third trimester in the study of Gallis et al [48] had a mean PHQ-9 score of 6.8 (range 0-27), which is more or less the same as the median PHQ-9 score of our study population (6, range 4-10).

#### PCS

At both 14 weeks of GA and at 32 weeks of GA, 33.3% (25/75) of participants reported catastrophizing; 4% (3/75) of this catastrophizing was found clinically depressed at enrollment, compared to 5.33% (4/75) at 32 weeks of GA. These numbers are higher than the 27.75% (72/242) of women who catastrophize at 19-21 weeks of GA and the 30.16% (73/242) of catastrophizing women at 34-37 weeks of GA reported by Olsson et al [41].

#### Strengths and Limitations

Our study is the first to show a significant change in psychological factors during pregnancies at risk for PIH. These results indicate a need for additional psychological support during pregnancies with this kind of complication.

Although the results of this study are promising, there are a number of limitations that should be considered. First, the generalizability of the results may be limited by the single-center design of the study. Second, the study results relied on self-reported data only. To complement the questionnaire data, clinical diagnostic interviews are required. Aside from circumventing shared method variance, this could have the additional advantage of offering the possibility to determine whether the study participants think the symptoms are pregnancy-related [48,49], as several symptoms of depression, that is, fatigue, appetite change, and sleep problems, have been shown to be associated with pregnancy. Third, the questionnaire data were gathered on a single-moment basis. It is possible that exceptional events influenced the respondents’ answers to the questionnaires. Third, there was a substantial number loss to follow-up (35/110, 20.53%). There is no information about the reason for loss to follow-up, which could have been of valuable information.

#### Recommendations for Further Research

It is known that depression rates tend to increase with each trimester, and the risk of generalized anxiety disorder and pain interference significantly increases over time during the third trimester [37,42,50]. In this study, 34.67% (26/75) of women based on the GAD-7 and 29.33% (22/75) of women based on the PHQ-9 had clinical depression. Future research needs to investigate how health care workers can help these women lower their depression rates. Second, pregnancy-specific anxiety is sometimes related to previous negative pregnancy experiences [51]. Additional research needs to be conducted to inform clinical decisions on whether specific mental programs are required for women with a history of PIH pregnancy. Next, this study shows an increase in anxiety and depression scores at 32 weeks of GA compared to the moment of enrollment in the PREMOM program and a decrease in catastrophizing, PCS scale. Data about this evolution in low-risk pregnancies are rare. It would be interesting to explore if the GAD-7, PHQ-9, and PCS scores of these pregnant women with PIH at the moment of enrollment and at 32 weeks of GA are significantly lower or higher compared to those of women with a no- or low-risk pregnancy. Finally, studies have already shown that participants with a high level of catastrophizing could benefit from cognitive behavioral therapy [51]. There are already existing behavioral therapies for non-pregnant women. Modifying those programs for pregnant women, and more specifically for pregnant women at risk for PIH, would have added value to the prenatal care program for them.

### Conclusions

The peripartum period has long been known to be associated with increased levels of stress and anxiety, which can be exacerbated by PIH. This study shows that pregnant women at risk for PIH have higher scores on the GAD-7 and PHQ-9 at 32 weeks of GA, compared to the moment of enrollment. The subscale scores of the PCS were all lower at 32 weeks of GA compared with 14 weeks of GA. Further research is recommended to explore if the GAD-7, PHQ-9, and PCS scores of these pregnant women with PIH at the moment of enrollment and at 32 weeks of GA are significantly lower or higher compared to women with a no- or low-risk pregnancy. The knowledge that follows from this research can help implement strategies to manage pregnant women at risk for PIH with their feelings of stress and anxiety.

## Abbreviations

BP: blood pressure
CG: control group
GA: gestational age
GAD-7: Generalized Anxiety Disorder-7 scale
PCS: Pain Catastrophizing Scale
PHQ-9: Patient Health Questionnaire-9
PIH: pregnancy-induced hypertension
PREMOM: Pregnancy Remote Monitoring
RM: remote monitoring
